# Species-independent bioassay for sensitive quantification of antiviral type I interferons

**DOI:** 10.1186/1743-422X-7-50

**Published:** 2010-02-26

**Authors:** Thomas Kuri, Matthias Habjan, Nicola Penski, Friedemann Weber

**Affiliations:** 1 Department of Virology, University of Freiburg, D-79008 Freiburg, Germany

## Abstract

**Background:**

Studies of the host response to infection often require quantitative measurement of the antiviral type I interferons (IFN-α/β) in biological samples. The amount of IFN is either determined via its ability to suppress a sensitive indicator virus, by an IFN-responding reporter cell line, or by ELISA. These assays however are either time-consuming and lack convenient readouts, or they are rather insensitive and restricted to IFN from a particular host species.

**Results:**

An IFN-sensitive, *Renilla *luciferase-expressing Rift Valley fever virus (RVFV-Ren) was generated using reverse genetics. Human, murine and avian cells were tested for their susceptibility to RVFV-Ren after treatment with species-specific IFNs. RVFV-Ren was able to infect cells of all three species, and IFN-mediated inhibition of viral reporter activity occurred in a dose-dependent manner. The sensitivity limit was found to be 1 U/ml IFN, and comparison with a standard curve allowed to determine the activity of an unknown sample.

**Conclusions:**

RVFV-Ren replicates in cells of several species and is highly sensitive to pre-treatment with IFN. These properties allowed the development of a rapid, sensitive, and species-independent antiviral assay with a convenient luciferase-based readout.

## Background

Type-I interferons (IFN-α/β) are potent cytokines that can be released from virtually all vertebrate cells following viral infection. They comprise a large number of IFN-α subspecies and a single IFN-β, and their actions reflect an important part of the innate immune system [1]. Upon infection, viruses are detected by one or several different pattern recognition receptors and production of IFN is induced. Newly synthesized IFNs are secreted in order to bind to their specific receptor (which is common for IFN-α and IFN-β) in an autocrine and paracrine manner. Receptor signaling via the Jak/Stat pathway leads to the up-regulation of a set of IFN-stimulated genes (ISGs), some of which having antiviral activity. As a consequence, neighbouring cells establish an antiviral state to prevent viral spread in the organism [2].

A wide variety of assays has been developed to determine the presence and activity of antiviral IFNs [3]. One type of assay is based on the upregulation of ISGs, either directly by measuring enzymatic ISG products [4], or indirectly by using cells containing a reporter gene under control of an IFN-responsive promoter. Often, the promoter of the Mx gene is used [5-8] due to the sensitivity and the low background expression of this ISG [9]. Although cell-line based ISG/reporter assays are rather convenient, a major drawback is their restriction to a particular host organism since IFNs bind to their receptor in a species-specific manner. In a similar vein, commercially available ELISAs are limited to a particular type of IFN and a single host species.

The historically oldest and still widely used assay to determine IFN activity are assays of antiviral activity. Here, IFN-mediated protection of cells is directly analyzed after infection with a sensitive challenge virus. The presence of IFNs is reflected by reduced cytopathic effects or diminished viral growth [10,11]. Some recent modifications of this assay take advantage of green fluorescent protein (GFP)-expressing viruses. These viruses allow to determine the reduction in virus titers by counting GFP-positive cells, either manually or by flow cytometry [12-14].

Rift Valley fever virus (RVFV) is a highly pathogenic member of the family *Bunyaviridae*. RVFV encodes a non-structural gene termed NSs which is mainly responsible for the pathogenicity of this virus [15,16]. Mutant RVFV lacking the NSs gene are thus highly sensitive to IFN-induced antiviral proteins such as MxA and PKR [17-20]. Here, we applied our recently developed reverse genetics system for RVFV [21] and replaced the NSs gene with the *Renilla *luciferase reporter gene, resulting in an attenuated, IFN-sensitive virus. We used this virus to establish a bioassay for quantification of IFN that combines the advantages of the classical antiviral assay with the convenience of luciferase reporter assays.

## Results and discussion

### Establishment of a luciferase-based antiviral IFN assay

**Figure 1:**
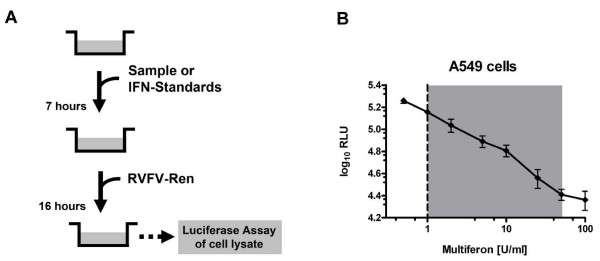
**Principle and evaluation of the RVFV-Ren antiviral assay**. (A) Schematic outline of the RVFV-Ren antiviral assay. (B) Dose-response curve in human A549 cells. A549 cells seeded in 96-well plates were treated with increasing doses of Multiferon for 7 hours before infection with 10,000 plaque forming units of RVFV-Ren. Sixteen hours later, cells were lysed and *Renilla *luciferase activity in cell lysates was determined. All measurements were performed in triplicate wells under standard conditions; shown are means ± *SD*. Both axes are plotted in logarithmic scale; the dotted vertical line indicates the threshold of sensitivity, based on the mean + 2*SD *of an untreated control run in triplicate; shaded area shows the linear range of the assay; RLU, relative light units.

Using our reverse genetics system [21], we generated the recombinant virus RVFV-Ren with the IFN antagonist NSs replaced by *Renilla *luciferase (see Materials and Methods). Luciferase expression by this virus was correlating with viral replication and remained stable over several passages (data not shown). We then tested the IFN sensitivity of RVFV-Ren in human A549 cells. Cells were seeded in 96-well microtiter plates and pre-treated with serial dilutions of standard IFN for 7 hours to allow the establishment of an antiviral state. Afterwards, cells were infected with RVFV-Ren at an MOI of 1 for 16 hours and *Renilla *luciferase activity was measured in cell lysates (Fig. 1A). To determine the linear range of the assay, IFN dilutions from 0.5 U/ml up to 100 U/ml were tested. A significant reduction of luciferase activity was observed using IFN concentrations from 1 U/ml on (Fig. 1B). The highest concentration of IFN in the linear range was 50 U/ml.

### Measurement of IFN in biological samples

**Figure 2:**
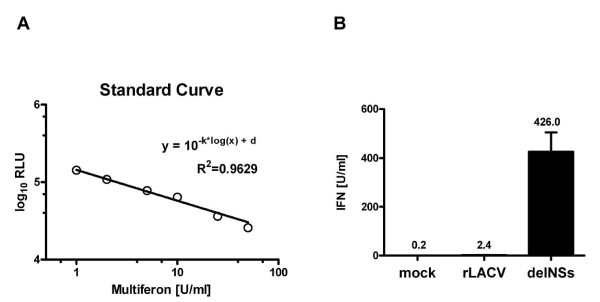
**Measurement of IFN in samples by the RVFV-Ren antiviral assay**. (A) Generation of the standard curve. All data points from Figure 1B which are above the sensitivity threshold and within the linear range of the assay (from 1-50 U/ml) are plotted. Non-linear regression analysis was performed to generate a standard curve and to compute the regression equation. Values for the slope (k) and the y-axis interception point (d) were -0.3988 and 5.159, respectively. The coefficient of regression (R^2^) indicates the linearity of detection. (B) Analysis of two samples with low and high concentration of IFN. Supernatants from human A549 cells infected with wt mutant La Crosse virus were first sterilized with β-propiolactone (see Methods section) and then analyzed for inhibition of RVFV-Ren. Sterilized supernatant from uninfected cells served as control (mock). The IFN content of all samples was extrapolated from the standard curve shown in (A). All measurements were performed in triplicate; columns show means ± *SD*, with actual values given above each column.

We used the RVFV-Ren assay to quantify IFN induction by two recombinant viruses which had been generated by our group. Wild-type (wt) La Crosse virus is able to suppress IFN induction, whereas a mutant virus upregulates the IFN-β gene [22]. Supernatants were taken from cells infected with these viruses and sterilized with β-propiolactone to destroy infectiousness. After removal of the disinfectant, undiluted and serially diluted samples of supernatants were subjected to the RVFV-Ren assay in parallel to the serial dilutions of standard IFN (see Fig. 1B). The IFN dilutions were used to create a standard curve by regression analysis (Fig. 2A) which, in turn, served to calculate IFN concentrations of the La Crosse virus supernatants. For cells infected with wt La Crosse virus, the activity of the undiluted supernatants was taken to determine the amount of type I IFN. For the mutant virus, we had to use the 1:10 dilution as the basis for the calculations since undiluted supernatants would have been out of the linear range of the assay. As expected, only little IFN was present in supernatants of cells infected with wt La Crosse virus, but substantial amounts were measured in the supernatant of mutant virus-infected cells (Fig 2B).

### Sensitive detection of murine and avian IFNs

**Figure 3:**
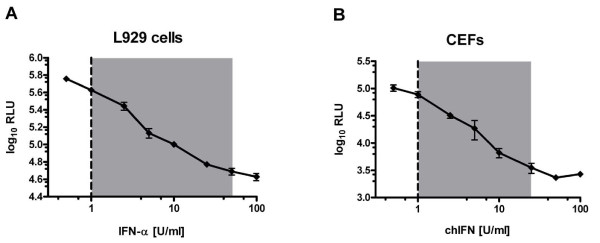
**Dose-response curves for mouse and chicken IFNs**. Murine L929 cells (A) or CEFs (B) seeded in 96-well plates were pre-treated for seven hours with increasing doses of pan-species IFN-α and chicken IFN (chIFN), respectively. Both assays were then carried out further as described for Fig. 1B. All measurements were performed in triplicate wells under standard conditions; shown are means ± *SD*. Both axes are plotted in logarithmic scale; the dotted vertical line indicates the threshold of sensitivity, based on the mean + 2*SD *of an untreated control run in triplicate; shaded area shows the linear range of the assay.

Since RVFV is able to infect a broad range of vertebrate hosts, we wanted to know whether the assay which was established for human IFN could be adapted to measure IFN from other species. To this aim, we infected L929 mouse cells and chicken embryo fibroblasts (CEFs), and tested the anti-RVFV-Ren activity of murine and avian IFN, respectively. As shown in Figure 3, RVFV-Ren was able to replicate in both cell lines and viral growth was inhibited in a dose-dependent manner by the species-compatible IFN. The sensitivity of the assay on L929 (Fig. 3A) as well as on CEFs (Fig. 3B) was comparable to human cells, with a linear range from 1 to 50 U/ml and from 1 to 25 U/ml, respectively.

### The RVFV-Ren assay measures type I IFNs

**Figure 4:**
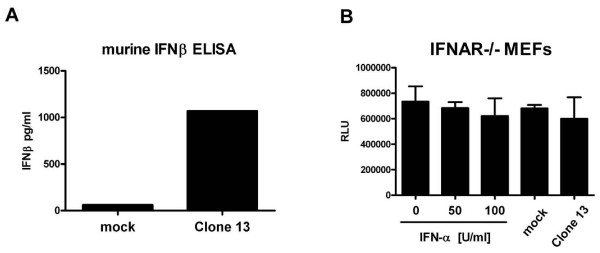
**RFVFV-Ren antiviral assay on cells lacking the type I IFN receptor**. Murine L929 cells were infected with Clone 13 at an MOI of 1, or left uninfected (mock). After 16 h of infection, supernatants were harvested and sterilized as described for Fig. 2B. (A) Determination of IFN-β using a commercial ELISA. Mean values from two measurements are shown. (B) Analysis for inhibition of RVFV-Ren on IFNAR-/- MEFs. Different doses of recombinant pan-species IFN-α were used as control. Measurements were performed in triplicate; columns show means ± SD.

Type I IFNs are the main, but not the only, cytokines produced during an innate immune response. We wanted to know whether other antiviral molecules, e.g. IFN-λ, could disturb our bioassay. To investigate this, we employed embryo fibroblasts from knockout mice lacking the receptor for type I IFNs (IFNAR-/- MEFs). Supernatants containing antiviral mouse cytokines were obtained by infecting L929 cells with the RVFV strain clone 13 [15,16], a virus mutant which in our experience is one of the strongest inducers of antiviral cytokines. Clone 13 upregulated an innate immune response including IFN-β, as expected (Fig. 4A). When we performed the bioassay on IFNAR-/- MEFs, the indicator virus RVFV-Ren was not inhibited by IFN-α, as expected, but also not by supernatants (Fig. 4B). This strongly indicates that it is type I IFNs which are causing the antiviral effect against RVFV-Ren.

Similar to the conventional virus inhibition or GFP-virus-based IFN bioassays, it can not be excluded *a priori *that material derived from e.g. infected dendritic cells or clinical samples may contain antiviral cytokines other than type I IFNs which could inhibit RVRV-Ren. Depending on the species material, IFNAR -/- MEFs, other mutant cell lines, acid treatment, or IFNAR-neutralizing antisera could be employed to verify that only type I IFNs are causing RVFV-Ren inhibition. However, in many cases supernatants from tissue cells are used, a system in which apparently type I IFNs are the dominant antiviral cytokine.

## Conclusions

Here we established a novel antiviral bioassay based on a recombinant RVFV encoding the gene for *Renilla *luciferase in place of the IFN-antagonistic NSs gene (RVFV-Ren). Growth of this virus is inhibited by IFN in a dose-dependent manner, which can easily be monitored by the measurement of luciferase activity in lysates of infected cells. Furthermore, IFN of at least three different species was reliably measured, indicating that RVFV-Ren not only infects cells of human or murine origin, as previously known, but also bird cells. The latter fact indicates that the RVFV-Ren assay could be used for other, less established species as well, e.g. bats. Our results show that these properties make this virus a useful tool for quantitative, species-independent measurement of IFN in biological samples, and that the use of luciferase and the 96-well plate format greatly facilitates readout. RVFV is classified as a BSL3 pathogen, and RVFV-Ren also needs to be handled under BSL3 conditions. The principle of our assay, however, namely the use of an IFN-sensitive virus expressing *Renilla *luciferase might as well be adapted for IFN-sensitive non-BSL3 viruses with a broad host range such as Vesicular stomatitis virus or Newcastle disease virus.

## Methods

### Cells and viruses

BHK-21, Vero E6 (ATCC; CRL-1586), 293T (ATCC; CRL-11268), human A549 (ATCC; CCL-185) cells, and murine L929 cells (ATCC; CCL-1) and IFNAR -/- MEFs (kindly provided from Jovan Pavlovic, University of Zurich, Switzerland) were cultivated in Dulbecco's modified Eagle's medium (DMEM) supplemented with 10% fetal calf serum (FCS; Biochrom AG) and antibiotics. Chicken embryo fibroblasts (CEF) were prepared from 10-day-old chicken embryos and maintained in DMEM with 2% chicken serum (Invitrogen), 8% FCS and antibiotics. Viruses used in this study were recombinant Rift Valley fever virus (RVFV) expressing *Renilla *luciferase (see below), RVFV Clone 13 [15,16], and recombinant La Crosse viruses expressing (wt) or lacking expression (mutant) of the NSs gene [22]. Virus titers were determined by standard plaque assay on Vero E6 cells.

### Interferons

Recombinant pan-species IFN-α (IFN-α B/D *Bgl*II) was purchased from PBL Biomedical Laboratories, and Multiferon, a mix of natural human IFN-α subtypes, was from Viragen. Recombinant chicken interferon (chIFN) was purified from *E. coli *and calibrated as described previously [23,24].

### Generation of recombinant RVFV expressing Renilla luciferase

We used our plasmid-based rescue system for generation of recombinant RVFV[21]. Expression of *Renilla *luciferase by RVFV-Ren was achieved by replacing the non-structural NSs gene on the genomic S segment with the *Renilla *gene. The corresponding S segment rescue plasmid pHH21-RVFV-vN_Ren was generated by inserting the *Renilla *luciferase open reading frame, amplified from plasmid pRL-SV40 (Promega), into the cloning site of pHH21-RVFV-vN_TCS [21]. RVFV-Ren was rescued by transfecting cocultures of 293T and BHK-21 cells in six-well plates with 0.5 μg of helper plasmids (pI.18-RVFV-L and pI.18-RVFV-N), together with 1 μg each of pHH21-RVFV-vL, pHH21-RVFV-vM, and pHH21-RVFV-vN_Ren using Nanofectin transfection reagent (PAA Laboratories). Supernatants containing recombinant viruses were collected 5 days post transfection and used to grow virus stock on Vero E6 cells. All RVFV rescues were performed under biosafety level (BSL) 3 conditions.

### Antiviral bioassay

Prior to measurement of IFN-containing samples, remaining virus was inactivated using β-propiolactone (Acros Organics) [11,25]. Briefly, supernatants were first incubated in the presence of 0.05% β-propiolactone in plastic dishes overnight at 4°C, and then at 37°C for 2 hours for hydrolysis of β-propiolactone.

Approximately 10,000 cells were seeded into each well of a 96-well microtiter plate and incubated overnight in a humified incubator at 5% CO_2 _and 37°C. Cells were then treated either with different dilutions of standard IFN or serial ten-fold dilutions of IFN-containing samples in 100 μl of growth medium for 7 hours. Subsequently, cell culture medium was removed and 10,000 plaque forming units of RVFV-Ren in 100 μl of infection medium (DMEM with 2% FCS and 20 mM HEPES, pH 7.3) were added per well. After 16 hours of further incubation, supernatants were removed and cells lysed in 50 μl of 1 × *Renilla *lysis buffer (Promega). Luciferase activity in 10 μl of cell lysate was measured using the *Renilla *luciferase assay system (Promega), according to the manufacturer's instructions.

## List of abbreviations

CEF: chicken embry fibroblast; DMEM: Dulbecco's modified Eagle's medium; GFP: green fluorescent protein; IFN: interferon; ISG: IFN-stimulated gene; MEF: mouse embryo fibroblast; RLU: relative light unit; RVFV: Rift Valley fever virus.

## Competing interests

The authors declare that they have no competing interests.

## Authors' contributions

TK carried out the antiviral assays and MH generated the RVFV-Ren virus. TK, MH and FW designed the study. NP produced recombinant chIFN and prepared CEFs. TK, MH, and FW were responsible for drafting and finalizing the manuscript. All authors read and approved the manuscript.
